# Accurate Quantum Chemical Spectroscopic Characterization of Glycolic Acid: A Route Toward its Astrophysical Detection

**DOI:** 10.1021/acs.jpca.2c01419

**Published:** 2022-04-06

**Authors:** Giorgia Ceselin, Zoi Salta, Julien Bloino, Nicola Tasinato, Vincenzo Barone

**Affiliations:** Scuola Normale Superiore, Piazza dei Cavalieri 7, I-56126, Pisa, Italy

## Abstract

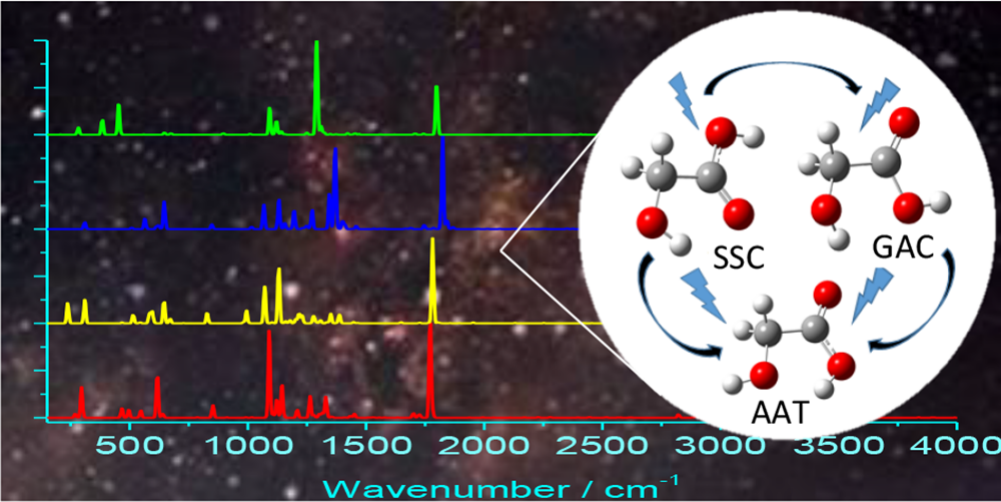

The first step to shed light on the abiotic synthesis of biochemical building blocks, and their further evolution toward biological systems, is the detection of the relevant species in astronomical environments, including earthlike planets. To this end, the species of interest need to be accurately characterized from structural, energetic, and spectroscopic viewpoints. This task is particularly challenging when dealing with flexible systems, whose spectroscopic signature is ruled by the interplay of small- and large-amplitude motions (SAMs and LAMs, respectively) and is further tuned by the conformational equilibrium. In such instances, quantum chemical (QC) calculations represent an invaluable tool for assisting the interpretation of laboratory measurements or even observations. In the present work, the role of QC results is illustrated with reference to glycolic acid (CH_2_OHCOOH), a molecule involved in photosynthesis and plant respiration and a precursor of oxalate in humans, which has been detected in the Murchison meteorite but not yet in the interstellar medium or in planetary atmospheres. In particular, the equilibrium structure of the lowest-energy conformer is derived by employing the so-called semiexperimental approach. Then, accurate yet cost-effective QC calculations relying on composite post-Hartree–Fock schemes and hybrid coupled-cluster/density functional theory approaches are used to predict the structural and ro-vibrational spectroscopic properties of the different conformers within the framework of the second-order vibrational perturbation theory. A purposely tailored discrete variable representation anharmonic approach is used to treat the LAMs related to internal rotations. The computed spectroscopic data, particularly those in the infrared region, complement the available experimental investigations, thus enhancing the possibility of an astronomical detection of this molecule.

## Introduction

Until the second half of the 20th century, the harsh conditions of the interstellar medium (ISM) were considered too hostile to host a chemistry capable of synthesizing polyatomic molecules. The detection of ammonia toward the center of our galaxy in 1968 through the observation of its inversion transitions^1^ radically changed that idea and set the birth of astrochemistry. Since then, more than 260 molecules have been detected in the interstellar medium or circumstellar shells, and about 70 species have been identified in extragalactic sources. Among the detected molecules, there are neutrals, radicals, and ions, with an increasing role being played by the so-called interstellar complex organic molecules (iCOMs),^2^ i.e., organic molecules containing more than six atoms, and, in particular, by those with a strong prebiotic character like, e.g., formamide, glycolaldehyde, and acetamide.^3^ The detection of iCOMs reveals that, despite the extreme physical conditions, a rich chemistry is at work in the universe, which is, however, not yet fully understood. Therefore, there is still much to be discovered about how iCOMs and prebiotic species are formed and how chemical complexity can evolve in both the ISM and planetary atmospheres. The starting point toward a satisfactory answer to these questions is the identification of the relevant species in the different astronomical environments, and then a reliable estimate of their abundances. Within this context, spectroscopy plays a crucial role because the observation of a molecule’s spectroscopic signature is the unequivocal proof for its presence. While most of the gas-phase species detected until now have been recognized via the ground-based observation of their rotational signature, the role played by infrared (IR) spectroscopy in retrieving the chemical composition of either planetary atmospheres or the ISM is expected to increase in the incoming years, also thanks to the spectrometers installed on airborne-based observatories, with the James Webb space telescope, launched last Christmas day, offering unique opportunities. Concerning prebiotic species, amides and organic acids can be considered to be the bricks for building biomolecules such as amino acids and nucleobases, which are on the path to the onset of life. In particular, a series of experiments has pointed out the pivotal role played by formamide, showing that its chemical processing in the presence of minerals could provide a one-pot route to the synthesis of a variety of nucleic acid bases and related compounds, such as low molecular weight amides and carboxylic acid derivatives.^4,5^ Despite the experimental evidence, and the fact that significant amounts of several organic acids have been measured in carbonaceous chondrites, up to now only formic (HCOOH) and acetic (CH_3_COOH) acids have been detected in the ISM. However, the analogies between the carboxylic and hydroxycarboxylic acids found in the Murchinson meteorite suggest similarities about their origin.^6^ Carboxylic acids are important intermediates in several metabolic processes taking place in cells for the production of energy and for the biosynthesis of primary and secondary metabolites; hence, understanding their sources and sinks in astronomical environments may help shed light on the mechanisms ruling the evolution toward chemical complexity in space or in planetary atmospheres. The first step in this direction is to ascertain the presence of carboxylic acids through astronomical observations, which in turn requires a precise spectroscopic characterization of the species most likely present, with rotational and vibrational signatures playing a central role.

Glycolic acid (CH_2_OHCOOH) is the hydroxyacid counterpart of acetic acid, obtained from the latter by replacement of one hydrogen atom of the methyl group with a hydroxyl moiety. At variance with acetic acid, no interstellar observation of glycolic acid has been reported until now, but potential formation pathways have been suggested, including high-energy proton irradiation of formamide in the presence of powdered meteorites^7^ and vacuum-UV processing of ice analogues containing H_2_O, NH_3_, and CH_3_OH.^8^ From a biological point of view, glycolic acid is involved in the glyoxylate cycle, an anabolic alternative of the Krebs cycle taking place in plants, bacteria, protists, and fungi.^9^ Alongside its potential astrochemical relevance, and its biological activity, the environmental role of glycolic acid is witnessed by its identification in atmospheric aerosols together with acetic, formic, pyruvic, and oxalic acids. Furthermore, about 3.6% of the organic content of aerosols in polluted regions of the troposphere is composed of glycolic acid.^10^

**Figure 1:**
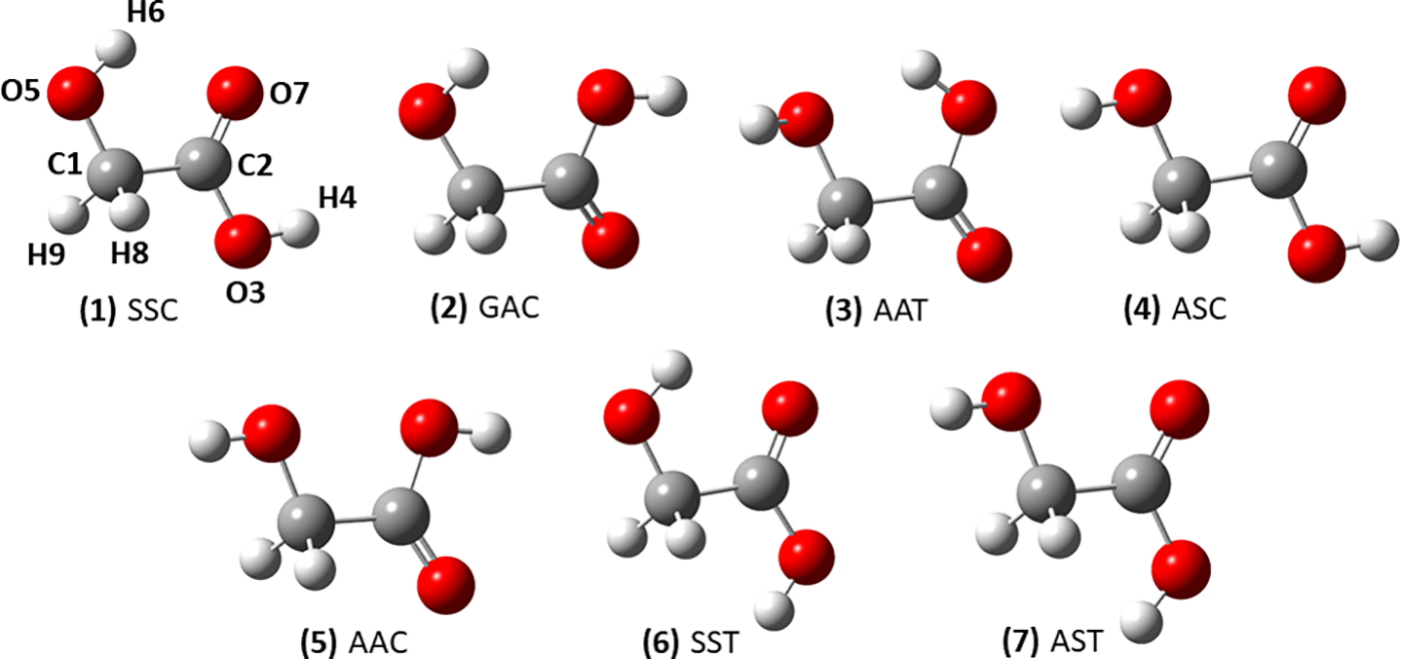
Molecular structures and labeling of glycolic acid conformers.

Over the years, glycolic acid has been the object of extensive research from both experimental and theoretical points of view, due to the remarkable interest of the potential energy landscape ruling its conformational dynamics. We will adopt the nomenclature proposed in refs (11 and 12), which employs the first letters of the minimum energy values of the following three dihedral angles: H–O–C–C (syn, anti, gauche), O–C–C=O (syn, anti), and O=C–C–H (cis, trans). Among the 12 possible conformers, the most refined computations (including those reported in the present paper) agree in forecasting the seven energy minima sketched in Figure 1. Four of these conformers are fully planar, thus belonging to the *C*_*S*_ point group, and one (GAC) is unequivocally nonplanar, thus lacking any symmetry. The situation is more involved for the AAT and AAC conformers, where the most refined computations agree in forecasting slightly nonplanar structures lacking any symmetry, but the barrier to planarity, when found, is so tiny that the zero-point vibrational energy (ZPVE) is largely sufficient to reach an effectively planar structure even at very low temperatures.

A pioneering study was carried out a long time ago by using ab initio self-consistent-field (SCF) computations and the 4-31G basis set with the aim of investigating the conformational energetic of α-hydroxycarbonyl species.^13^ For glycolic acid, the conformational energy profiles of four conformers were computed on nonoptimized structures. Geometry optimizations were carried out a few years later for six conformers by assuming a planar skeleton, arriving at the conclusion that the SSC conformer is the most stable one but pointing out some inconsistencies between the obtained structures and the available experimental data.^14^ More recently, Jensen et al. optimized the geometries of eight different conformers at the MP2/6-31G(d,p) level of theory and worked out relative energies from the CCSD(T)/6-31G(d,p), MP2/6-311++G(2d,2p), and MP2/cc-pVQZ methods. In that work, a basis set dependence of the predicted planarity of the heavy atom skeleton was reported for some conformers, and two of them resulted in being essentially isoenergetic, differing only for the value of the HOCC torsional angle involving the alcoholic hydroxyl group.^15^

From the experimental side, the microwave spectrum of the SSC conformer (see Figure 1) was first investigated in the early 1980s by Blom and Bauder,^16,17^ who measured the rotational spectra of the main isotopologue as well as of the D, ^13^C, and ^18^O isotopic species, determined the dipole moment components (μ_*a*_ = 1.913, μ_*b*_ = 0.995 D), and used the retrieved rotational constants to obtain its substitution structure. In parallel, a reinvestigation of the microwave spectra led to the revision of the dipole moment components (μ_*a*_ = 1.95, μ_*b*_ = 1.02 D), the refinement of the substitution structure, and the measurement of the rotational constants in the first and second vibrationally excited levels of the lowest-energy normal mode.^18^ About 15 years later, Godfrey et al. performed ab initio computations at the MP2/6-31G(d,p) level of theory to drive the interpretation of the free-jet microwave spectrum and were able to assign the rotational spectrum of the AAT conformer^19^ (see Figure 1). A few years ago, the pure rotational spectrum was reinvestigated in the 115–318 GHz region; the transitions among all the vibrational states up to 400 cm^–1^ were measured and analyzed, and the frequency of the lowest fundamental vibration was estimated to be around 98 cm^–1^.^20^

Several research efforts were devoted to explore the vibrational properties of glycolic acid. An early work by Günthard’s group focused on the IR spectra of the main isotopologue of the SSC conformer and 11 of its isotopologues in an argon matrix.^21^ In a subsequent work, the same research group succeeded in the identification of the AAT conformer, again trapped in an Ar matrix, obtained by IR-induced isomerization of the SSC isomer, and studied the photoconversion kinetics as well.^22^ A third conformer (GAC, see Figure 1), trapped in a low-temperature noble gas matrix, was observed in the early 2000s by Fourier transform IR spectroscopy (FTIR), and the experimental results were compared with harmonic frequencies computed at the MP2/aug-cc-pVDZ level.^11,23^ About 10 years ago, the SST conformer (see Figure 1) was generated by near-IR laser excitation and its FTIR spectra recorded in both noble gas or N_2_ matrixes were analyzed.^24^ The analysis of the near-IR region around 1.4 μm allowed the identification of bands attributed to the two OH-stretching overtones for the SSC, AAT, and GAC conformers, while the corresponding fundamentals remained unresolved in the noble gas matrix. However, the two OH-stretching frequencies of the SSC conformer, which coalesce in a single absorption at 3561 cm^–1^ in an Ar matrix, give rise to a well-defined doublet with maxima at 3574 and 3540 cm^–1^ in solid N_2_, thus pointing out a strong and unsymmetrical environmental effect of the matrix on the intramolecular hydrogen-bond framework and hence on the OH-stretching frequencies. Very recently, further work by FTIR spectroscopy in a noble gas matrix allowed for the identification of two bands, at 10182 and 10116 cm^–1^, attributed to the second overtone of the acidic and alcoholic hydroxyl groups, respectively.^12^ The region between 13 300 and 13 420 cm^–1^, featuring the signals stemming from the third OH-stretching overtones, which were reported at 13 373 and 13 351 cm^–1^,^25^ was recorded in the gas phase by cavity ring-down spectroscopy. A previous FTIR investigation in the gas phase failed to resolve the OH-stretching fundamental bands but resulted in the measurement of a number of combination bands.^26^ The high overtone-induced isomerization of glycolic acid in a low-temperature argon matrix was also studied by using Raman spectroscopy that allowed the characterization of the SSC, GAC, and AAT conformers.^27^ Next, kinetic measurements led to the proposal of a detailed model involving direct SSC ⇌ AAT and SSC ⇌ GAC isomerizations, which was employed to derive isomerization rate constants. The close pair of acidic and alcoholic OH-stretching fundamental bands has been resolved only in 2020 by gas-phase Raman spectroscopy in a supersonic jet, allowing their assignment at 3586 and 3578 cm^–1^, respectively.^28^

Despite the huge research efforts devoted to the investigation of the structural and spectroscopic features of glycolic acid, several questions remain to be solved in order to achieve the knowledge required for its detection in the ISM or planetary atmospheres. First, an accurate molecular structure is still lacking and the available data only refer to the SSC conformer. Second, microwave and millimeter/submillimeter wave spectroscopic studies have been able to detect only the SSC and AAT forms, whereas IR and Raman experiments have led to the identification of the GAC and SST conformers too. Third, vibrational frequencies in the gas phase have been measured only for the most stable conformer (SSC) by Raman spectroscopy. This implies that quantitative information is still lacking for the IR intensities of the SSC conformer, while for the remaining conformers the reported frequencies can be affected by the presence of the matrix, with the above discussion suggesting that matrix effects can be particularly strong for OH-stretching frequencies.

On these grounds, the present work is devoted to a detailed investigation of the structure and relative stability of the low-energy conformers of glycolic acid, together with their rotational and infrared spectroscopic features by means of state-of-the-art quantum chemical calculations, with the aim of providing new accurate data capable of boosting deeper spectroscopic investigations and/or assisting the interpretation of observational data.

## Methods

Structural, energetic, and spectroscopic properties of the conformers of glycolic acid reported in Figure 1 were computed following a well-consolidated procedure^29−31^ relying on the use of composite schemes based on the coupled-cluster ansatz including single, double, and a perturbative estimate of triple excitations (CCSD(T)) and on hybrid force fields obtained by combining equilibrium and harmonic properties obtained by composite methods with anharmonic contributions computed using density functional theory (DFT).^32,33^ According to the available literature, the double-hybrid B2PLYP^34^ and revDSD-PBEP86^35^ functionals in conjunction with suitable triple-ζ basis sets can be recommended for the purpose in view of their good performance in the prediction of geometries and rotational–vibrational spectroscopic parameters.^29,36−39^ On the basis of previous experience, the B2PLYP double hybrid functional^34^ was used in conjunction with the maug-cc-pVTZ-dH basis set (obtained by removing d functions on hydrogen atoms from the maug-cc-pVTZ basis set^40^), whereas the jun-cc-pVTZ basis set^41^ was preferred for the revDSD-PBEP86 functional. Dispersion-correlation effects were always taken into account by the Grimme’s D3 scheme^42^ employing the Becke–Johnson damping function.^43^ In the following, these two computational levels will be referred to as B2 and rDSD, respectively.

For each conformer, geometry optimizations were first carried out, followed by evaluation of analytical Hessians. Best estimates for the equilibrium structures of the different conformers were obtained employing the so-called “cheap” composite scheme^44^ (ChS hereafter) in which all the structural parameters are first optimized at the CCSD(T) level of theory in conjunction with the cc-pVTZ basis set.^45,46^ On top of this, contributions for the complete basis set (CBS) extrapolation and for the effects of core–valence (CV) correlations were applied. The CBS extrapolation was carried out by using the *n*^–3^ two-point equation^47^ applied to the values of structural parameters obtained by second-order Møller–Plesset (MP2)^48^ perturbation theory employing cc-pVTZ and cc-pVQZ basis sets.^45,46^ Core–valence correlation contributions were obtained from the differences between the values calculated at the MP2 level in conjunction with the cc-pCVTZ basis set^49^ by correlating all electrons and within the frozen-core approximation. On the basis of several benchmark studies, the method is expected to predict bond lengths and valence angles with an accuracy within 2 mÅ and 0.1–0.2°, respectively.^50−52^

The ChS was also used to obtain best estimates of the harmonic vibrational frequencies of the different conformers of glycolic acid and to characterize all the stationary points identified on the conformational potential energy surface (PES). Best estimates of harmonic IR intensities of each normal mode *i* within the ChS, *I*_*i*_^ChS^, were computed according to the following expression:
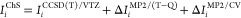
1where the first term on the rhs is the harmonic intensity at the CCSD(T)/cc-pVTZ level, while the second and the third terms account for the enlargement of the basis set and the contribution from the core–valence correlation, respectively. The former contribution is obtained from the difference between MP2 values computed with the cc-pVQZ and cc-pVTZ basis sets, while the latter contribution is the difference between intensities calculated at the MP2/cc-pCVTZ level by correlating all and only valence electrons, respectively. Although representing an empirical approximation, this approach has been shown to provide reliable predictions.^53,54^

Best estimates of the electronic energies were computed on geometries optimized at the B2 level, by using the jun-ChS variant of the cheap scheme,^31,55^ which provides an improved description of noncovalent interactions without excessive increase of the computational cost by replacing the cc-pVnZ basis sets with the corresponding jun-cc-pVnZ partially augmented counterparts,^41^ while keeping the same core-correlation contribution.

Spectroscopic parameters beyond the rigid-rotor–double-harmonic approximation were derived within the framework of second-order vibrational perturbation theory (VPT2)^56−58^ by using the computed equilibrium geometries, harmonic properties, and anharmonic force constants. Cubic and semidiagonal quartic force constants and second- and third-order derivatives of the dipole moment were obtained through numerical differentiation of B2 analytical Hessian matrices and first-order derivatives of the dipole moments, respectively. To overcome the problem of possible resonances plaguing the expressions of vibrational energies and transition moments, resonant terms were removed from the perturbative summations, thus providing the corresponding deperturbed quantities. The neglected contributions were then reintroduced in a subsequent step, the so-called generalized VPT2 (GVPT2), which employs the deperturbed energies and the relevant interaction matrix elements to set up the proper interaction Hamiltonian, whose eigenvalues are the perturbed energy levels, and the corresponding eigenvectors are used to project the deperturbed transition moments.^59^ Anharmonic thermodynamic functions were computed by the so-called hybrid degeneracy-corrected second-order perturbation theory (HDCPT2), which provides accurate yet resonance-free vibrational energies.^59^

Finally, some of the conformers of glycolic acid appeared to be not well-described as semirigid molecules, with a few vibrational modes behaving as large-amplitude motions (LAMs) for which the perturbative treatment resulted in unphysically large anharmonic corrections (vide infra). In order to overcome this issue, the LAMs were treated separately by means of one-dimensional (1D) discrete variable representations (DVR), with the couplings between the LAM and the small-amplitude motions (SAMs) being neglected. In detail, the large-amplitude torsion was described as the distance (in mass-weighted Cartesian coordinates) between structures obtained by a relaxed scan (i.e., optimizing all the other degrees of freedom at each point) of the dihedral angle providing the overwhelming contribution to this mode in steps of 10°. The details of the procedure are given in previous studies,^50,60^ and successful applications have been reported for the methyl internal rotation of the methyl-cyclopropenyl cation^61^ and the nitrogen inversion in nitroxide radicals.^62^

Coupled-cluster computations were performed with the CFOUR program,^63^ whereas MP2 and DFT calculations were carried with the Gaussian 16 suite of programs,^64^ whose built-in GVPT2 engine was also employed to evaluate anharmonic contributions.^59,65^

## Results and Discussion

In the following, the PES of the glycolic acid ruling the interconversion between the different conformers is discussed first. Then, the attention is focused on the molecular structures of the minima identified on the PES, with the derivation of the equilibrium geometry for the most stable SSC conformer. Next, the predicted rotational spectroscopic parameters are presented, and finally, the IR spectra simulated beyond the double-harmonic approximation are discussed.

### Conformational Landscape

**Figure 2:**
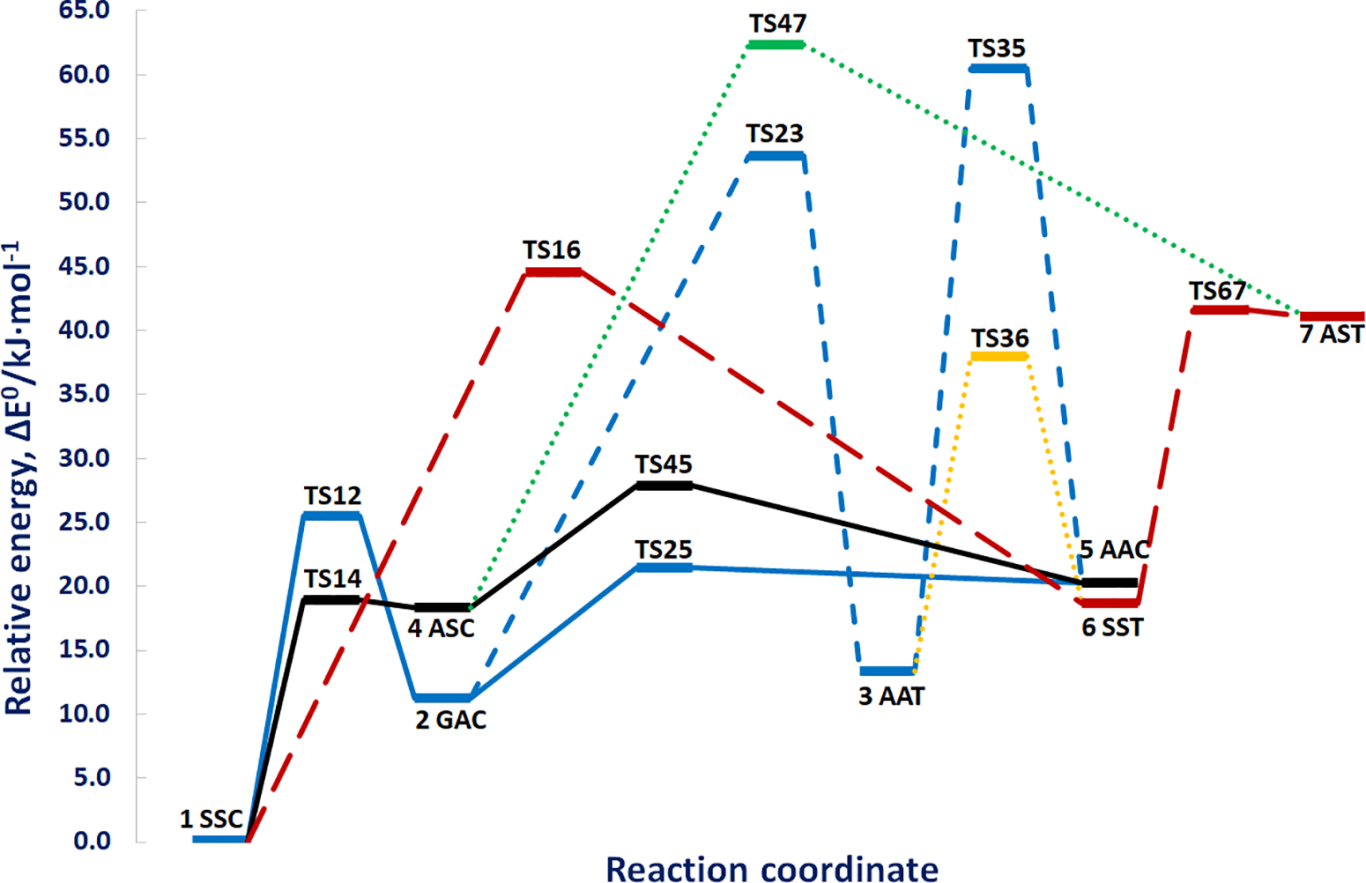
Conformational PES of glycolic acid. Relative ground-state energies (Δ*E*_0_ in kJ mol^–1^) are obtained from jun-ChS electronic energies and B2 anharmonic ZPVEs.

The relative electronic (Δ*E*_el_) and ground-state (Δ*E*_0_ = Δ*E*_el_ + anharmonic ZPVE) energies of the different conformers of glycolic acid and of the transition states (TSs) ruling their interconversion are listed in Table 1, whereas the conformational PES is shown in Figure 2. The close similarity between the B2 and jun-ChS energies gives further support to the use of B2 geometries for more refined single-point energy computations of TSs. In this connection, we point out that the structures of all the energy minima will be discussed in a specific section, whereas the Cartesian coordinates of all the stationary points optimized at the B2 level and the imaginary frequencies of the TSs are given in the Supporting Information.

**Table 1:** Relative Electronic Δ*E*_el_ and Ground-State Δ*E*_0_ Energies (kJ mol^–1^) of the Stationary Points on the Conformational PES of Glycolic Acid

species	Δ*E*_el_^B2^^a^	Δ*E*_el_^jChS^^b^	Δ*E*_0_^jChS:B2^^c^
SSC	0.00	0.00	0.00
GAC	10.54	11.46	11.23
AAT	13.31	13.48	13.39
ASC	19.42	19.49	18.34
AAC	20.45	21.12	20.28
SST	20.26	19.24	18.67
AST	44.50	43.50	41.14
TS12	24.29	25.41	25.52
TS14	20.55	20.83	18.90
TS16	50.69	49.37	44.56
TS23	58.79	58.25	53.68
TS25	20.46	21.21	21.43
TS35	67.06	66.36	60.49
TS36	37.64	37.95	37.96
TS45	27.98	28.54	27.83
TS47	69.54	68.22	62.33
TS67	45.10	44.48	41.62

a Electronic energies at the B2 level.

b Electronic energies at the jun-ChS level.

c Electronic energies at the jun-ChS level corrected by B2 anharmonic ZPVEs.

In agreement with all previous theoretical and experimental investigations, the SSC (**1**) conformer is the most stable. Three major paths emerge from this global minimum, which lead, respectively, to the ASC (**4**), GAC (**2**), and SST (**6**) conformers. Kinetically, the lowest TS14 transition state would make the isomerization of SSC (**1**) to ASC (**4**) the most favorable one. However, the barrier for the reverse path is very low (0.5 kJ mol^–1^), with this suggesting that fast relaxation of ASC (**4**) should prevent its experimental detection. The second possibility is the conversion of SSC (**1**) to GAC (**2**) via TS12, with a barrier of about 25 kJ mol^–1^. The GAC (**2**) conformer lies about 11 kJ mol^–1^ above the global minimum, and assuming a Boltzmann distribution among the conformers, its relative abundance should be around 1% at room temperature, with this suggesting that the experimental observation is made possible only by matrix trapping. Indeed, as stated by Halasa et al.,^24^ no repopulation of the initial most stable SSC (**1**) conformer was observed upon near-IR excitation of the higher-energy forms of the compound isolated in solid Ar. Conversely, the detection of the GAC (**2**) conformer through rotational spectroscopy, which is in principle possible, appears more challenging in view of its relatively small dipole moment (vide infra). It is then possible to form AAC (**5**) from GAC (**2**) by overcoming TS25, but the forward barrier is relatively high (about 21 kJ mol^–1^), and especially, the reverse barrier is just 1 kJ mol^–1^. Therefore, formation of the AAC (**5**) conformer appears unlikely, and above all, it would immediately be converted to GAC (**2**), in agreement with the lack of any experimental detection.

According to Ahokas et al.,^27^ upon near-IR excitation in a N_2_ matrix, the most stable SSC (**1**) conformer is converted solely into the SST (**6**) counterpart. However, the energy barrier (TS16) ruling the direct interconversion between these two conformers is quite high, suggesting that there should be another path open for this transformation. In the investigation performed by Halasa et al.,^24^ it was further shown that, even though the GAC (**2**) conformer can be produced directly via the near-IR excitation of SSC (**1**), the next higher-energy conformer, namely, AAT (**3**), was effectively generated only upon further excitation of the primary GAC (**2**) photoproduct with another near-IR photon. Inspection of Figure 2 shows that the energy difference between TS23 (53.7 kJ mol^–1^) and TS12 (25.6 kJ mol^–1^) can be easily attributed to the extra photon necessary for the generation of the SST (**6**) conformer. Finally, the least stable AST (**7**) conformer has not yet been detected. Indeed, even though it can be generated starting from either ASC (**4**) or SST (**6**) conformers, the energy barriers ruling the reverse processes are exceedingly low, making the production of the AST (**7**) conformer very unlikely.

In summary, the energy barriers ruling the conversions of the ASC (**4**) conformer to SSC (**1**), AAC (**5**) to GAC (**2**), and AST (**7**) to SST (**6**) are so low that the conformers ASC (**4**), AAC (**5**), and AST (**7**) should not be experimentally detectable. As a consequence these three conformers will not be analyzed in detail in the following. While the remaining four conformers [SSC (**1**), GAC (**2**), AAT (**3**), and SST (**6**)] can be characterized by vibrational spectroscopy (and all of them have actually been identified), the situation is different for rotational spectroscopy. Indeed, the SST (**6**) conformer, lying about 20 kJ mol^–1^ above the global minimum SSC (**1**), could not be detected in a sufficient amount, whereas the small population and the low dipole moment of the GAC (**2**) conformer (see below) might generate spectral lines of exceedingly low intensity. As a consequence, only conformers SSC (**1**) and AAT (**3**) appear safely detectable by microwave spectroscopy, in full agreement with the available experimental data.

### Structural Properties

As a first step, the equilibrium geometry of the SSC conformer has been determined by using the semiexperimental (SE) approach. Indeed, while the ground-state rotational constants of different isotopologues were measured by several researchers,^17−19^ to the best of our knowledge, only effective ground-state or substitution structures were determined.^17,18^ It is well-known, however, that the accuracy of these geometries can be limited because vibrational effects are not taken into proper account. The SE method rectifies this situation, and thus, it is the best way to determine accurate equilibrium structures for nontrivial (i.e., larger than three atoms) molecules in the gas phase.^66^ According to the SE approach,^67^ the equilibrium geometry is obtained by a nonlinear least-squares fit of the semiexperimental rotational constants of a set of isotopologues. These are in turn obtained by correcting the experimentally determined rotational constants (usually of the ground vibrational state) with vibrational contributions evaluated theoretically:
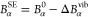
2where α = *A*, *B*, *C* denotes the principal axis of inertia, *B*_α_^SE^ and *B*_α_^0^ are the SE and ground-state rotational constants, respectively, and Δ*B*^vib^ represents the vibrational correction, whose evaluation requires semidiagonal cubic force constants.^66,68^

The SE of SSC glycolic acid has been obtained by using the ground-state rotational constants of the main isotopic species^20^ and those of the CH_2_OH^13^COOH, ^13^CH_2_OHCOOH, CH_2_OHCO^18^OH, CH_2_OHC^18^OOH, CH_2_^18^OHCOOH, CHDOHCOOH, CH_2_ODCOOH, CH_2_OHCOOD, and CH_2_ODCOOD isotopologues,^17^ with vibrational contributions evaluated in the framework of the VPT2 model at the B2 level of theory. The structural refinement has been performed by employing the MSR (molecular structure refinement) program,^69^ which provided also a detailed error analysis.^70^ The resulting SE equilibrium geometry of the SSC conformer is reported in Table 2 together with the theoretical structures evaluated in the present work. As can be seen, the SE equilibrium geometry appears well-determined, with errors within 1 mÅ and 0.2° for bond lengths and valence angles, respectively. The only exception is the O_3_—H_4_ distance, whose statistical error is slightly larger, around 3 mÅ, but still acceptable.

**Table 2:** Semiexperimental and Theoretical Equilibrium Geometries and Equilibrium Rotational Constants for the SSC Conformer of Glycolic Acid^a^

	*r*_e_^SE^^b^	ChS	CCSD(T)^c^	B2	rDSD	rDSD + NL^d^
C_1_—C_2_	1.5050_5_(6_7_)	1.5052	1.5114	1.5078	1.5096	1.5051
C_2_—O_3_	1.3385_7_(5_5_)	1.3379	1.3446	1.3442	1.3437	1.3397
O_3_—H_4_	0.965_8_(3_8_)	0.9641	0.9678	0.9688	0.9686	0.9667
C_1_—O_5_	1.3993_5_(3_8_)	1.3975	1.4039	1.4035	1.4034	1.3995
O_5_—H_6_	0.9647_2_(4_0_)	0.9630	0.9665	0.9671	0.9672	0.9598
C_2_—O_7_	1.204_7_(1_1_)	1.2032	1.2098	1.2086	1.2089	1.2060
C_1_—H_9_	1.0903_7_(3_8_)	1.0918	1.0946	1.0926	1.0951	1.0927
∠(C_1_C_2_O_3_)	112.59_6_(7_0_)	112.53	112.57	112.48	112.41	112.33
∠(C_2_O_3_H_4_)	106.8_6_(2_3_)	107.08	106.35	107.34	107.06	107.06
∠(C_2_C_1_O_5_)	110.84_5_(3_6_)	110.81	110.69	111.18	111.03	110.73
∠(C_1_O_5_H_6_)	106.52_9_(4_4_)	106.64	105.40	106.77	106.46	106.59
∠(C_1_C_2_O_7_)	123.98_5_(3_4_)	124.08	124.03	123.58	123.60	123.26
∠(C_2_C_1_H_9_)	108.00_1_(5_7_)	108.12	107.97	108.23	108.18	108.24
δ(O_7_C_2_C_1_H_9_)	121.96_3_(7_0_)	121.90	122.01	121.97	121.92	121.74
*A*_e_	10793.000	10801.210	10701.919	10718.270	10701.571	10748.898
*B*_e_	4096.842	4102.927	4072.963	4063.057	4066.505	4091.652
*C*_e_	3025.087	3029.188	3005.304	3000.863	3001.792	3019.143

a Bond lengths in angstroms, angles in degrees, and rotational constants in megahertz.

b Semiexperimental equilibrium geometry; values in parentheses are one standard deviation in the units of the last significant digits.

c CCSD(T)/cc-pVTZ.

d rDSD-corrected by nano-LEGO (ref (71)).

When the SE geometry is compared to the theoretical estimate obtained at the ChS level, a very good agreement is noted, with the maximum differences being 2 mÅ for bond lengths and 0.2° for all the valence angles. The CCSD(T)/cc-pVTZ geometry, on the other hand, does not show any improvement over the structures obtained by using the double-hybrid functionals. In fact, on average, bond lengths are systematically overestimated by 4 mÅ, with errors as large as 6 mÅ, while for bond angles, the mean absolute deviation is 0.3°, thus being very similar to (strictly speaking, slightly worse than) the results delivered by both the B2 and rDSD levels of theory. Conversely, more accurate structures are obtained by improving rDSD geometrical parameters by means of the recently proposed nano-LEGO (from the Latin for “put together”) tool,^71^ which employs the so-called template molecule approach (TMA) to correct the starting geometrical parameters by the differences between semiexperimental and computed values for suitable fragments (synthons) of the molecular system at hand. Then, the geometrical parameters not available in any reference fragment are improved by the linear regression approach (LRA)^68^ in which systematic errors for bond lengths and valence angles of different pairs and triplets of atom types are corrected by linear regressions, whose parameters were derived from a large database of semiexperimental equilibrium geometries. In the specific case of glycolic acid, the structural parameters of the HO–CH_2_C=O and =C–OH moieties have been refined by using glycolaldehyde and formic acid, respectively, as templating synthons, whereas the interfragment angles have been corrected through the LRA. As shown in Table 2, the nano-LEGO-corrected geometry closely approaches the accuracy of the ChS composite scheme with a significantly reduced computational cost: bond distances are reproduced with an absolute average error of 1.5 mÅ (to be compared with 1.3 mÅ for the ChS method), and the error on valence angles is well within 0.3°, with the only exception being the C_1_C_2_O_7_ angle.

The ChS equilibrium geometries of the remaining conformers of glycolic acid, whose SE structure cannot be determined due to the lack of experimental data, are collected in Table 3. On the basis of the results obtained for the SSC conformer, as well as the available literature, their average accuracy is expected to be around 2 mÅ for bond distances and 0.2° for bond angles.

**Table 3:** Equilibrium Geometries of the GAC, AAT, SST, ASC, and AAC Conformers of Glycolic Acid at the ChS Level^a^

*r*_e_^ChS^	GAC	AAT	SST	ASC	AST	AAC
C_1_—C_2_	1.5108	1.5177	1.5141	1.5073	1.5161	1.5085
C_2_—O_3_	1.3480	1.3358	1.3425	1.3506	1.3564	1.3358
O_3_—H_4_	0.9639	0.9664	0.9608	0.9635	0.9603	0.9645
C_1_—O_5_	1.4028	1.4171	1.3937	1.4011	1.3982	1.4056
O_5_—H_6_	0.9582	0.9559	0.9646	0.9564	0.9568	0.9565
C_2_—O_7_	1.1993	1.1965	1.1974	1.1951	1.1889	1.2027
C_1_—H_8_	1.0948	1.0898	1.0943	1.0929	1.0954	1.0913
C_1_—H_9_	1.0854	1.0892	1.0943	1.0929	1.0954	1.0926
∠(C_1_C_2_O_3_)	111.92	115.32	116.49	109.54	113.56	114.13
∠(C_2_O_3_H_4_)	106.71	107.80	110.31	106.38	110.65	105.89
∠(C_2_C_1_O_5_)	114.68	108.98	110.69	108.86	109.00	111.40
∠(C_1_O_5_H_6_)	108.49	109.94	106.06	108.26	108.49	108.22
∠(C_1_C_2_O_7_)	124.66	121.79	122.23	126.76	125.45	122.06
∠(C_2_C_1_H_8_)	106.33	107.21	108.45	107.33	107.66	106.24
∠(C_2_C_1_H_9_)	107.62	107.64	108.45	107.33	107.66	106.09
δ(C_1_C_2_O_3_H_4_)	177.67	–0.67	0.00	180.00	0.00	–179.49
δ(O_3_C_2_C_1_O_5_)	25.01	4.94	180.00	180.00	180.00	–7.01
δ(C_1_C_2_O_5_H_6_)	–44.19	–164.39	0.00	180.00	180.00	177.42
δ(O_5_C_1_C_2_O_7_)	–157.76	–175.55	0.00	0.00	0.00	173.80
δ(O_7_C_2_C_1_H_8_)	77.72	63.06	–121.57	–122.24	–121.89	50.89
δ(O_7_C_2_C_1_H_9_)	–37.53	–53.89	121.57	122.24	121.89	–63.68

a Bond lengths in angstroms; angles in degrees.

### Rotational Spectroscopy

The ChS equilibrium geometries of the different conformers of glycolic acid provide equilibrium rotational constants, which, corrected for the vibrational contributions evaluated at the B2 level, represent reliable estimates of the ground-state rotational constants (referred to as ChS:B2 in the following). These are listed in Tables 4 and 5 for the SSC and AAT conformers, together with the available experimental values. The ChS:B2 rotational constants reproduce very well the experimental results for both the SSC and AAT conformers, with an average percentage error around 0.17%. The CCSD(T)/cc-pVTZ, B2, and rDSD models give close, albeit slightly worse, results, which underestimate the experimental values by about 0.8% and 0.7% for the SSC and AAT conformers, respectively. A remarkable agreement between theoretical estimates and experimental data is apparent also for the quartic centrifugal distortion constants of the SSC conformer, particularly those obtained from ChS with an average error of about 1% and a maximum deviation of 5% for the δ_*K*_ parameter. The remaining methods underestimate the quartic centrifugal distortion constants by about 3.5% with maximum errors between 7.7% (revDSD) and 9.1% (CCSD(T)), thus confirming the expected accuracy.^36,72^ Concerning the AAT conformer, a striking deviation of about 50% is observed for the Δ_*K*_ centrifugal distortion parameter, irrespective of the level of theory employed. On the basis of the results obtained for the SSC conformer, this difference appears too large and might be ascribed to the difficulty in measuring the rotational transitions of the AAT species, which have been derived from a fit including only 95 lines, to be compared with the 2050 transitions employed for the most stable conformer.^20^ It is also noteworthy that, at variance with the other quartic centrifugal distortion parameters, the experimental determination of Δ_*K*_ appears very challenging. Indeed, the value of 3.60 kHz reported by Kisiel et al.^20^ is very different from that obtained by Godfrey et al.^19^ (4.89 kHz), which is in much better agreement with the computed counterpart (5.40 kHz). Concerning the sextic centrifugal distortion constants, due to the lack of experimental data for the AAT species, comparison between theory and experiment is possible only for the SSC conformer. The computed values of the Φ_*JK*_, Φ_*K*_, ϕ_*JK*_, and ϕ_*K*_ parameters show errors within 10% from the experimental counterparts, as expected on the basis of previous benchmark studies.^37,73^ However, a discrepancy of about 60% in opposite directions is observed for Φ_*J*_ and ϕ_*J*_. A possible explanation for this behavior may be rooted in the small values, especially of Φ_*J*_, that make their precise determination a difficult task. For this reason, it would be interesting to perform a new fit of the assigned rotational transitions using the theoretical estimates of the sextic centrifugal distortion constants as initial guesses or even by fixing Φ_*J*_ and ϕ_*J*_ to the computed values. It is finally noteworthy that also the computed components of the ground-state dipole moment are in very good agreement with the experimental values.^18^

**Table 4:** Rotational Spectroscopic Parameters for the SSC Conformer of Glycolic Acid^a^

	ChS:B2^b^	CCSD(T):B2^c^	B2	rDSD:B2^d^	exptl^e^
*A*_0_	10700.00	10600.71	10617.06	10600.36	10696.09254(20)
*B*_0_	4055.86	4025.90	4015.99	4019.44	4051.032059(80)
*C*_0_	2998.40	2974.52	2970.07	2971.00	2994.662720(79)
Δ_*J*_	0.813	0.797	0.798	0.796	0.817090(54)
Δ_*JK*_	3.162	3.055	3.108	3.092	3.214417(81)
Δ_*K*_	5.671	5.617	5.586	5.533	5.68401(78)
δ_*J*_	0.210	0.206	0.206	0.206	0.2096352(71)
δ_*K*_	2.487	2.386	2.396	2.421	2.62367(16)
Φ_*J*_ × 10^4^			0.27	0.33	0.67(11)
Φ_*JK*_ × 10^2^			–0.649	–0.589	–0.5954(54)
Φ_*KJ*_			–0.016	–0.017	–0.2259(15)
Φ_*K*_			0.044	0.044	0.04314(97)
ϕ_*J*_ × 10^4^			0.452	0.464	0.284(14)
ϕ_*JK*_ × 10^2^			–0.221	–0.206	–0.2449(55)
ϕ_*K*_			0.016	0.016	0.01511(37)
|μ_*a*_|	1.91	1.93	1.95	1.92	1.95(4)
|μ_*b*_|	1.00	0.84	0.97	0.97	1.02(4)

a Rotational constants in megahertz, quartic centrifugal distortion constants in kilohertz, sextic centrifugal distortion constants in hertz, and dipole moment in debyes. Values refer to Watson’s *A*-reduction Hamiltonian in the *I*^*r*^ representation.

b Equilibrium rotational constants from the ChS equilibrium geometry corrected for vibrational contributions at the B2 level.

c CCSD(T)/cc-pVTZ equilibrium rotational constants corrected for vibrational contributions at the B2 level.

d Equilibrium rotational constants at the rDSD level corrected for B2 vibrational contributions.

e Rotational and centrifugal distortion constants from ref (20) and dipole moment components from ref (18). Values in parentheses are standard errors in units of the last significant digits.

**Table 5:** Rotational Spectroscopic Parameters of the AAT Conformer of Glycolic Acid^a^

	ChS:B2^b^	CCSD(T):B2^c^	B2	rDSD:B2^d^	exptl^e^
*A*_0_	10292.49	10214.28	10219.00	10215.40	10273.5661(60)
*B*_0_	4224.50	4172.12	4175.60	4181.24	4207.0082(18)
*C*_0_	3052.64	3025.69	3023.71	3024.94	3048.49166(51)
Δ_*J*_	0.866	0.887	0.858	0.863	0.88445(98)
Δ_*JK*_	3.214	3.004	3.278	3.264	3.147(29)
Δ_*K*_	5.401	5.538	5.317	5.254	3.60(21); 4.89(94)^f^
δ_*J*_	0.247	0.251	0.240	0.245	0.25041(51)
δ_*K*_	2.823	2.757	2.808	2.832	2.644(16)
Φ_*J*_ × 10^3^			–0.536		n.a.
Φ_*JK*_ × 10^2^			–0.689		n.a.
Φ_*KJ*_			–0.017		n.a.
Φ_*K*_			0.042		n.a.
ϕ_*J*_ × 10^3^			–0.226		n.a.
ϕ_*JK*_ × 10^2^			–0.586		n.a.
ϕ_*K*_ × 10^2^			0.479		n.a.
|μ_*a*_|	4.68	4.50	4.67	4.65	n.a.
|μ_*b*_|	1.07	0.96	0.98	0.99	n.a.
|μ_*c*_|	0.16	0.57	0.46	0.32	n.a.

a Rotational constants in megahertz, quartic centrifugal distortion constants in kilohertz, sextic centrifugal distortion constants in hertz, and dipole moment in debyes. Values refer to Watson’s *A*-reduction Hamiltonian in the *I*^*r*^ representation.

b Equilibrium rotational constants from the ChS equilibrium geometry corrected for vibrational contributions at the B2 level.

c CCSD(T)/cc-pVTZ equilibrium rotational constants corrected for vibrational contributions at the B2 level.

d Equilibrium rotational constants at the rDSD level corrected for B2 vibrational contributions.

e From ref (20). Values in parentheses are standard errors in units of the last significant digits. n.a.: not available.

f From ref (19).

The predicted spectroscopic parameters of the GAC and SST conformers are reported in Table 6, where, for the rotational and quartic centrifugal distortion constants, the estimates obtained at both the ChS:B2 and B2 levels have also been improved by a scaling procedure^61^ starting from the results obtained for the SSC conformer:
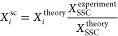
3where *X*_*i*_^sc^ and *X*_*i*_^theory^ are, respectively, the scaled and theoretical spectroscopic constants of conformer *i* (i.e., SSC, GAC, or SST) and *X*_SSC_^experiment^ is the same parameter measured experimentally for the SSC conformer. On the basis of the predictions for the SSC and AAT conformers, as well as of the available literature, the expected accuracy is better than 0.1–0.2%, for rotational constants, 5–10% for the quartic, and 10–15% for the sextic centrifugal distortion parameters.

**Table 6:** Rotational Spectroscopic Parameters for the GAC and SST Conformers of Glycolic Acid^a^

	GAC		SST	
	ChS:B2	sc-ChS:B2	ChS:B2	sc-ChS:B2
*A*_0_	10111.50	10107.81	10567.73	10563.88
*B*_0_	4131.43	4126.51	4072.37	4067.52
*C*_0_	3028.43	3024.66	2996.53	2992.79
Δ_*J*_	1.018	1.043	0.823	0.843
Δ_*JK*_	4.362	4.511	2.727	2.820
Δ_*KJ*_	7.417	7.547	5.596	5.694
δ_*J*_	0.215	0.219	0.216	0.220
δ_*K*_	4.047	4.431	2.376	2.601
Φ_*J*_ × 10^4^	4.243		0.518	
Φ_*JK*_ × 10	–0.123		–0.052	
Φ_*KJ*_	–0.221		–0.020	
ϕ_*J*_ × 10^3^	–0347		0.058	
ϕ_*JK*_ × 10^2^	0.145		–0.200	
ϕ_*JK*_	–0.058		1.190	
|μ_*a*_|	0.03		0.50	
|μ_*b*_|	1.53		3.25	
|μ_*c*_|	0.96		0.00	

a Rotational constants in megahertz, quartic centrifugal distortion constants in kilohertz, sextic centrifugal distortion constants in hertz, and dipole moment in debyes. Values refer to Watson’s *A*-reduction Hamiltonian in the *I*^*r*^ representation; ChS:B2 refers to ground-state rotational constants obtained from ChS equilibrium rotational constants corrected with B2 vibrational contributions and B2 centrifugal distortion constants; sc-ChS:B2 refers to scaled theoretical values (see main text for details).

### Vibrational Spectroscopy

**Figure 3:**
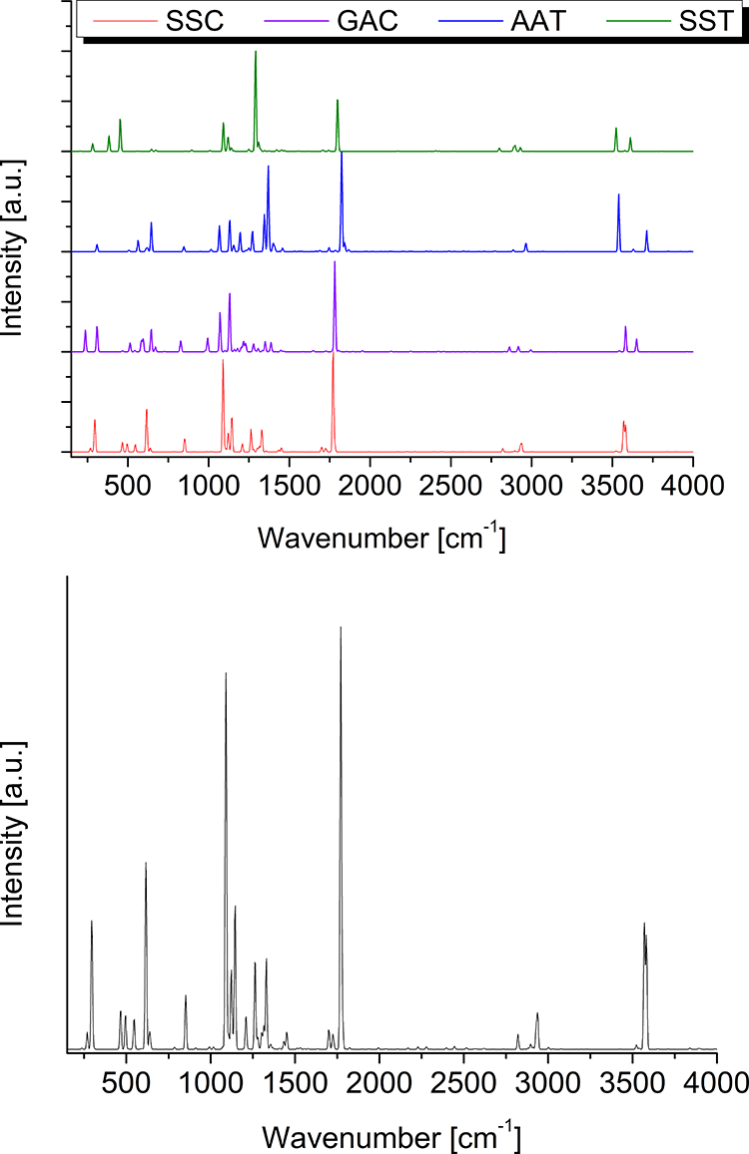
Computed infrared spectra of the SSC, GAC, AAT, and SST conformers of glycolic acid (upper panel) and overall simulated infrared spectrum of glycolic acid obtained by considering the relative abundances of the different conformers at 300 K (lower panel). The simulated stick spectrum has been convoluted with a Gaussian function with a half-width at half-maximum of 5 cm^–1^.

The infrared spectra of the SSC, GAC, AAT, and SST conformers in the gas phase have been simulated beyond the double-harmonic approximation using a hybrid approach^33^ in which the best estimates of the harmonic vibrational frequencies have been integrated with anharmonic corrections obtained at the B2 level of theory. The resulting simulations are shown in Figure 3, where the total spectrum, obtained by Boltzmann averages of the contributions of the four conformers, is also presented. Before discussing the results, it should be recalled that most of the experiments devoted to investigate the vibrational spectra of glycolic acid have been carried out using the matrix isolation technique (mostly in noble gases, but also in N_2_),^11,12,21−24,27^ with gas-phase data being limited to the OH-stretching overtone regions,^25,26^ and only recently a portion of the gas-phase spectrum of the SSC conformer has been measured by Raman spectroscopy in a supersonic jet.^28^

Let us start our discussion with the SSC conformer, not only because it is the most stable one but, more importantly, because the corresponding gas-phase experimental data can represent a benchmark for the adopted methodology, which, in general, is expected to predict fundamental transition frequencies with an average error of around 5 cm^–1^ (and maximum errors within 10 cm^–1^)^50,54^ and IR intensities with an accuracy of a few kilometers per mole.^50,53^ As already mentioned, the SSC conformer belongs to the *C*_*S*_ symmetry point group, and its vibrations can be classified, in terms of symmetry species, as 14*A*′ ⊕ 7*A*″. The predicted fundamental frequencies of this conformer are collected in Table 7, where they are compared to the available experimental data taken from different sources, and anharmonic IR intensities are also reported. The table provides two sets of anharmonic data: columns four and five refer to full GVPT2 computations, whereas the columns six and seven collect wavenumbers and intensities obtained by restricting the GVPT2 treatment to normal modes ν_1_–ν_20_ and excluding ν_21_. The latter vibration actually corresponds to the torsion around the C–C bond, partially hindered by the presence of the hydrogen bond between the alcoholic hydrogen and the oxygen of the carbonyl moiety. By looking at the full GVPT2 calculation, it is apparent that the anharmonic correction results in an unusual positive contribution of 20 cm^–1^ that, even if not dramatic, is diagnostic of a large-amplitude vibration. For this reason, a 1D anharmonic DVR treatment has been applied to the lowest-frequency normal mode, ν_21_, obtaining a fundamental frequency of 92 cm^–1^. This can be compared to the value of 152 cm^–1^, reported in the very first experimental study carried out in an Ar matrix, which, however, appears too high and has never been confirmed by any subsequent IR or Raman spectroscopic investigation. On the other hand, the wavenumber of 98 cm^–1^ estimated for the ν_21_ fundamental from the analysis of the rotational spectra in excited vibrational states^20^ closely matches the DVR prediction. Inspection of Table 7 shows that removal of the contributions from the ν_21_ vibration from the GVPT2 model has little effect on the frequencies of the remaining small-amplitude modes, suggesting that most of the couplings between SAMs and the single LAM are indeed small. Only ν_7_ and ν_20_ are notably affected, and in both cases the fundamental frequencies obtained from the reduced-dimensionality GVPT2 calculations are closer to the experimental values than those stemming from the full-GVPT2 model, thus giving further support to the reliability of the adopted approach. Comparison between the experimentally measured transition frequencies and their ChS:B2 counterparts shows an overall good agreement, with a mean absolute deviation of 4.4 cm^–1^, coherent with the expected accuracy of the approach.^50,54^ It is gratifying that the frequencies of the two O–H-stretchings, that have attracted the attention of vibrational spectroscopic studies due to their close energies until their recent resolution,^28^ are well-reproduced by the calculations. It is noteworthy that also the computed splitting between the ν_4_ fundamental and the ν_9_ + ν_12_ combination band (14 cm^–1^) is in remarkable agreement with the experimental counterpart (15 cm^–1^), with the computed IR intensity of the combination band (13.69 km mol^–1^) being non-negligible. The close reproduction of the positions of the (ν_1_/ν_2_) and (ν_4_/ν_9_ + ν_12_) pairs of closely spaced bands without the involvement of any strong resonance (as claimed in ref (28) especially for the second pair) gives further support to the robustness of the GVPT2 engine and the accuracy of the hybrid ChS:B2 quantum chemical model.

**Table 7:** Harmonic (ω^ChS^, *I*^ChS^) and Anharmonic (ν^ChS:B2^, *I*^ChS:B2^) Wavenumbers (cm^–1^) and Intensities (km mol^–1^) for the Fundamental Vibrational Bands of the SSC Conformer of Glycolic Acid^a^

mode	ω^ChS^	*I*^ChS^	ν^ChS:B2^ (full)^b^	*I*^ChS:B2^ (full)^b^	ν^ChS:B2^ (RD)^c^	*I*^ChS:B2^ (RD)^c^	exptl
ν_1_	3763	85.58	3595	69.80	3582	75.99	3586^d^
ν_2_	3751	76.03	3562	64.21	3569	62.21	3578^d^
ν_3_	3043	26.87	2938	26.88	2938	17.23	2929^d^
ν_4_	1802	284.12	1771	238.27	1771	237.04	1782^d^
ν_5_	1501	11.87	1452	10.57	1451	10.82	1451.6^e^
ν_6_	1477	0.32	1434	0.79	1433	1.16	1438.7^e^
ν_7_	1354	123.38	1311	101.50	1330	48.56	1332.2^e^
ν_8_	1296	26.75	1264	26.10	1263	56.67	1264.8^e^
ν_9_	1183	134.01	1144	102.01	1144	98.21	1143.3^e^
ν_10_	1122	227.08	1086	275.16	1090	195.93	1090.1^e^
ν_11_	870	30.19	852	25.10	852	26.61	854.1^e^
ν_12_	649	17.16	642	9.88	639	10.81	638^e^
ν_13_	474	25.13	467	17.25	467	23.71	467.7^e^
ν_14_	278	7.66	267	9.06	269	8.81	269.5^f^
ν_15_	3078	6.88	2925	8.80	2929	8.57	2921^d^
ν_16_	1264	0.05	1229	0.38	1233	0.07	1231.4^e^
ν_17_	1042	1.32	1016	1.16	1016	1.19	1018.8^e^
ν_18_	640	108.56	619	96.31	616	97.91	618^e^
ν_19_	508	11.22	496	21.72	496	20.94	495.2^e^
ν_20_	342	78.61	307	73.70	296	72.37	280.6^f^
ν_21_	95	10.44	113	6.68	92^g^	0.00^g^	152^f^, 98^h^

a Modes 1–14 have *A*′ symmetry, and modes 15–21 have *A*″ symmetry.

b Full-dimensionality GVPT2 treatment.

c Reduced-dimensionality GVPT2 treatment (ν_21_ excluded).

d Raman gas-phase measurement from ref (28).

e IR Ar matrix measurement from ref (12).

f IR, noble gas matrix measurement from ref (21).

g Anharmonic 1D DVR treatment.

h Estimated for the gas phase in ref (20).

The only not fully satisfactory result concerns mode ν_20_, whose frequency has been reported at 281 cm^–1^ from noble gas matrix IR spectra,^21^ while calculations place it at 296 cm^–1^ and no other significant contributions are expected in this spectral region. Possible explanations for the disagreement can be related to a misinterpretation of the experimental spectrum or to a large shift of the free-molecule frequency induced by the noble gas matrix. All in all, comparison between computed and experimental data confirms the expected accuracy of the ChS:B2 computational protocol, i.e., a mean absolute deviation around 5 cm^–1^ for fundamental transitions, thus giving further support to the application of this method to the remaining conformers of glycolic acid for which no gas-phase IR spectroscopic characterization has been possible until now.

Harmonic and anharmonic fundamental wavenumbers and intensities for the SST, AAT, and GAC conformers are reported in Tables 8, 9, and 10, respectively, together with the available experimental data measured by trapping the molecule in low-temperature matrix and inducing photoisomerization by IR irradiation. Among these minor conformers, the most complete set of experimental data is available for the AAT conformer, while only a few bands have been identified for the GAC and SST species and, for the former, some appear as shoulders of more prominent absorptions.

**Table 8:** Harmonic (ω^ChS^, *I*^ChS^) and Anharmonic (ν^ChS:B2^, *I*^ChS:B2^) Wavenumbers (cm^–1^) and Intensities (km mol^–1^) of the Fundamental Vibrational Bands for the SST Conformer of Glycolic Acid^a^

mode	ω^ChS^	*I*^ChS^	ν^ChS:B2^ (full)^b^	*I*^ChS:B2^ (full)^b^	ν^ChS:B2^ (RD)^c^	*I*^ChS:B2^ (RD)^c^	exptl^d^
ν_1_	3796	67.43	3612	55.74	3611	55.91	3580^e^
ν_2_	3726	84.89	3547	75.71	3544	74.22	n.a.
ν_3_	3016	38.77	2803	12.18	2802	12.09	n.a.
ν_4_	1833	230.52	1800	213.46	1800	212.93	1798^e^
ν_5_	1502	8.81	1451	6.57	1450	6.53	n.a.
ν_6_	1466	0.94	1433	0.00	1421	0.00	n.a.
ν_7_	1327	409.92	1283	296.03	1282	303.35	1295^e^
ν_8_	1299	43.65	1263	103.36	1262	95.51	n.a.
ν_9_	1182	2.25	1152	1.97	1152	2.03	n.a.
ν_10_	1129	158.03	1094	91.37	1098	103.56	1099^e^
ν_11_	876	1.04	857	0.74	856	0.73	n.a.
ν_12_	662	15.18	648	10.85	652	14.05	n.a.
ν_13_	473	0.52	461	1.74	465	0.53	n.a.
ν_14_	285	27.77	276	27.10	277	27.77	n.a.
ν_15_	3048	10.16	2899	14.74	2899	20.66	n.a.
ν_16_	1279	0.16	1249	0.26	1251	0.17	n.a.
ν_17_	1039	0.85	1013	0.66	1013	0.65	n.a.
ν_18_	575	0.69	557	0.47	560	0.59	n.a.
ν_19_	472	129.94	463	64.40	451	106.35	510^e^
ν_20_	383	58.39	334	101.77	339	71.85	n.a.
ν_21_	107	4.84	116	4.84	107^f^	4.95^f^	n.a.

a Modes 1–14 have *A*′ symmetry, and modes 15–21 have *A*″ symmetry.

b Full-dimensionality GVPT2 treatment.

c Reduced-dimensionality GVPT2 treatment (ν_21_ excluded).

d n.a.: not available

e From IR N_2_ matrix measurement (ref (24)).

f Harmonic value.

**Table 9:** Harmonic (ω^ChS^, *I*^ChS^) and Anharmonic (ν^ChS:B2^, *I*^ChS:B2^) Wavenumbers (cm^–1^) and Intensities (km mol^–1^) of the Fundamental Vibrational Bands for the AAT Conformer of Glycolic Acid^a^

mode	ω^ChS^	*I*^ChS^	ν^ChS:B2^ (full)^b^	*I*^ChS:B2^ (full)^b^	ν^ChS:B2^ (RD)^c^	*I*^ChS:B2^ (RD)^c^	exptl^d^
ν_1_	3862	59.85	3681	46.73	3677	52.05	3672^e^
ν_2_	3717	150.69	3529	132.44	3526	137.49	3474^e^
ν_3_	3107	8.63	2953	6.54	2960	11.40	2976^f^
ν_4_	3055	15.68	2963	13.22	2959	6.97	2952^f^
ν_5_	1835	275.98	1799	178.04	1800	178.64	1806^e^
ν_6_	1504	8.36	1460	6.72	1460	7.88	1448^g^
ν_7_	1430	90.11	1396	76.18	1393	92.18	1388^g^
ν_8_	1382	300.13	1351	110.56	1353	91.68	1360^g^
ν_9_	1266	18.98	1188	0.00	1231	6.31	n.a.
ν_10_	1254	2.44	1290	6.70	1224	6.06	1198^g^
ν_11_	1157	111.18	1078	79.49	1109	76.19	1136^g^
ν_12_	1086	44.53	1047	0.00	1055	36.74	1059^g^
ν_13_	1040	5.31	1023	2.43	1018	4.94	n.a.^g^
ν_14_	860	11.59	840	10.58	841	10.90	846^g^
ν_15_	622	71.77	635	1.80	645	22.60	653^g^
ν_16_	660	14.18	609	9.58	582	29.79	n.a.^f^
ν_17_	571	23.46	518	52.88	558	23.40	559^h^
ν_18_	512	3.84	503	3.08	504	3.74	503^h^
ν_19_	312	17.93	301	15.75	302	17.45	309^g^
ν_20_	127	74.86	–189	996.2	55^i^	74.86^j^	n.a.
ν_21_	84	49.33	19.1	56.20	84^j^	49.33^j^	n.a.

a All the normal modes are ordered by decreasing wavenumber due to the lack of any symmetry.

b Full-dimensionality GVPT2 treatment.

c Reduced-dimensionality GVPT2 treatment (ν_20_ and ν_21_ excluded).

d n.a.: not available.

e Average of measurements of refs (22) (IR, Ar matrix), (24) (IR N_2_ matrix), and (27) (Raman, Ar matrix).

f From Raman Ar matrix measurement (ref (27)).

g From IR Ar matrix measurement (ref (22)).

h Average of measurements of refs (22) (IR, Ar matrix) and (27) (Raman, Ar matrix).

i Anharmonic 1D-DVR treatment.

j Harmonic value.

**Table 10:** Harmonic (ω^ChS^, *I*^ChS^) and Anharmonic (ν^ChS:B2^, *I*^ChS:B2^) Wavenumbers (cm^–1^) and Intensities (km mol^–1^) of the Fundamental Vibrational Bands for the GAC Conformer of Glycolic Acid^a^

mode	ω^ChS^	*I*^ChS^	ν^ChS:B2^ (full)^b^	*I*^ChS:B2^ (full)^b^	ν^ChS:B2^ (RD)^c^	*I*^ChS:B2^ (RD)^c^	exptl^d^
ν_1_	3829	51.36	3649	45.41	3643	46.30	3648^e^
ν_2_	3766	85.13	3581	74.07	3582	73.87	3568^e^
ν_3_	3141	3.66	2995	5.92	3002	5.22	n.a.
ν_4_	3021	24.59	2862	9.78	2859	12.40	2875^f^
ν_5_	1814	283.12	1781	255.25	1782	258.39	1785^e^
ν_6_	1494	4.45	1447	4.46	1446	4.08	n.a.
ν_7_	1425	73.39	1399	46.55	1388	64.12	n.a.
ν_8_	1374	46.37	1347	34.70	1354	29.21	n.a.
ν_9_	1336	16.59	1303	0.00	1311	0.00	n.a.
ν_10_	1244	40.69	1196	20.03	1214	22.01	n.a.
ν_11_	1172	195.43	1133	164.17	1130	168.41	n.a.
ν_12_	1103	88.70	1071	105.10	1072	87.16	1080^g^
ν_13_	1013	15.42	991	2.94	992	13.46	998^g^
ν_14_	847	26.03	827	24.72	828	25.92	826^g^
ν_15_	674	70.12	676	27.04	647	41.28	n.a.
ν_16_	601	87.30	578	54.44	584	59.46	596^f^
ν_17_	528	24.17	511	26.24	518	27.48	511^f^
ν_18_	472	6.27	462	12.98	464	7.47	467^f^
ν_19_	309	67.84	269	51.03	309^h^	67.84^h^	n.a.
ν_20_	237	61.46	224	78.59	224	61.46	n.a.
ν_21_	81	4.40	74	5.16	81^h^	4.40^h^	n.a.

a All the normal modes are ordered by decreasing wavenumber due to the lack of any symmetry.

b Full-dimensionality GVPT2 treatment.

c Reduced-dimensionality GVPT2 treatment (ν_19_ and ν_20_ excluded).

d n.a.: not available.

e Average of measurements of refs (12) (IR, Ar matrix), (11) (IR, Ar matrix), and (27) (Raman, Ar matrix).

f From Raman Ar matrix measurement (ref (27)).

g Average of measurements of refs (12) (IR, Ar matrix) and (11) (IR, Ar matrix).

h Harmonic value.

As expected, the general features of the computed spectrum of the SST conformer resemble those of the SSC counterpart due to the same symmetry (*C*_*S*_) and backbone conformation. Once again, inclusion of anharmonic contributions at the VPT2 level for ν_21_ leads to a significant anharmonic blue shift. However, contrary to the case of the SSC conformer, this mode includes non-negligible contributions by several internal coordinates so that a one-dimensional treatment becomes questionable. The lack of any experimental information and the closeness of harmonic and DVR results for the SSC conformer led us to retain the harmonic wavenumber for ν_21_. The agreement with the few experimental data available for this conformer is definitely worse than the expected one (and actually found for the SSC conformer), but this is probably related to the increased experimental challenges and the non-negligible matrix effects mentioned above.

The last two conformers (GAC and AAT) lack any symmetry and involve an intramolecular hydrogen bridge between the two hydroxyl moieties, whose internal rotations are quite flat. From a technical point of view, the anharmonic description of the OH rotation of the CH_2_OH group (ν_20_ for AAT, which becomes ν_19_ in GAC) by a fourth-order polynomial expansion of the potential energy appears problematic. Since this LAM is dominated by a single internal coordinate (the CCOH torsional angle mentioned above), we carried out its DVR treatment in the case of the AAT species for which the VPT2 anharmonic correction was completely nonphysical. Finally, the ν_21_ mode of the AAT conformer is not well-approximated by just the C–C torsion, and as a consequence, the one-dimensional DVR description was not attempted.

Coming to comparison with the available experimental results, a fair agreement is observed for the AAT conformer, even though a difference as large as 52 cm^–1^ is noted for the ν_2_ vibration corresponding to the stretching of the acidic O–H group, which can be possibly due to a shift induced by the interaction with the Ar matrix environment. Actually, such an effect is present also in the most stable SSC conformer for which the two O–H-stretching vibrations have been measured at 3586 and 3578 cm^–1^ in the gas phase, while they give rise to two bands at 3574 and 3540 cm^–1^ in N_2_ matrix and coalesce in an unresolved bundle centered at 3561 cm^–1^ in Ar matrix.^24^ A similar shift can be also noted by comparing the computed and measured O–H-stretching frequencies of the GAC conformer, even though it seems smaller than in the case of AAT, a finding that is consistent with the fact that species with a larger dipole moment are expected to be more stabilized by matrix effects.^23^ Finally, it should also be noted that AAT seems the most flexible conformer of glycolic acid. Indeed, as can be seen in Table 9, the perturbative approach coupled to the fourth-order Taylor expansion of the potential energy used to account for the anharmonicity resulted in a completely nonphysical correction of about 300 cm^–1^ for ν_20_, whose computed anharmonic wavenumber becomes negative. This LAM, which corresponds to the torsion of the alcoholic O–H group, was then described with a 1D-DVR treatment, obtaining an anharmonic wavenumber of 55 cm^–1^.

## Conclusions

State-of-the-art quantum chemical computations show that the small, but highly flexible, glycolic acid has seven energy minima, which are structurally related to rotations around the C–C and the two C–O single bonds. Characterization of the saddle points ruling the interconversion between different pairs of conformers suggests the relaxation of three conformers to the four most stable ones, which should be the only ones amenable to experimental investigations. This prediction is in full agreement with the available data from vibrational spectroscopy experiments, either in the gas phase or in inert matrix. Furthermore, anharmonic computations in the framework of generalized second-order vibrational perturbation theory integrated by one-dimensional quasi-variational treatments of large-amplitude motions lead to remarkable agreement with experiment for the most stable conformer, which is the only one characterized experimentally in the gas phase. The agreement is less satisfactory for some vibrations of the other conformers, possibly due to the role played by matrix effects.

Only two conformers have been characterized by microwave spectroscopy, whereas the lower stability and smaller dipole moments of the other two conformers have not yet allowed their microwave characterization. In this respect, the availability of rotational spectra for several isotopologues of the most stable conformer has allowed the determination of a very accurate equilibrium structure by means of the semiexperimental approach. The remarkable agreement between this structure and the corresponding rotational parameters with those obtained by a composite quantum chemical approach (cheap scheme) allows for the prediction of accurate parameters for all four low-energy conformers mentioned above. In particular, some of the computed sextic centrifugal distortion constants call for a re-examination of the available experimental fittings.

Coming to the astrochemical implications, the potential formation of this prebiotic molecule in the ISM is likely to be mediated by icy-dust grains, to be then released in the gas phase during the warm-up phase. However, a careful analysis of the feasibility of this route deserves a dedicated investigation, which is out of the scope of the present work. The computed data suggest that the SSC, GAC, AAT, and SST conformers might be worthy of detection. However, only the most stable SSC conformer could be possibly detected by radio astronomical searches, whereas the most promising experimental strategy for the detection of the remaining conformers is offered by IR spectroscopy. Unfortunately, the experimental data collected until now are barely usable for the interpretation of astronomical data. On the one side, the available measurements for the minor conformers have been performed at low temperature in matrix, which can cause frequency shifts with respect to the gas-phase unperturbed vibrations. On the other hand, even though some regions of the vibrational spectrum have been recorded in the gas phase for the SSC conformer, Raman spectroscopy has been employed for this purpose, and hence, no information on IR transition intensities is available. Therefore, the outcomes of the present study provide further information which could be of significant help in the search for glycolic acid in extraterrestrial environments.

## Supporting Information Available

Table containing the Cartesian coordinates of all the B2PLYP-D3BJ/maug-cc-pVTZ-dH stationary points together with the corresponding imaginary frequencies of the transition states (PDF)
