# Supplementary material for: Accurate Quantum Chemical Spectroscopic Characterization of Glycolic Acid: A Route Toward its Astrophysical Detection
Source: J Phys Chem A. 2022 Apr 6;126(15):2373–87. doi: 10.1021/acs.jpca.2c01419 (PMC9036519; doi:10.1021/acs.jpca.2c01419)
Supplement: Supplementary file 1 — jp2c01419_si_001.pdf [file jp2c01419_si_001.pdf]

Supporting Information: Accurate Quantum Chemical Spectroscopic Characterization of Glycolic Acid: A Route Toward its Astrophysical Detection[†]

Giorgia Ceselin, Zoi Salta, Julien Bloino, Nicola Tasinato,* and Vincenzo Barone*

Scuola Normale Superiore, Piazza dei Cavalieri 7, I-56126, Pisa, Italy

E-mail: nicola.tasinato@sns.it; vincenzo.barone@sns.it

Phone: +39 050 509134. Fax: +39 050 509160

[†]A footnote for the title

Table S1 Cartesian Coordinates (Å) of minima and transition states on the conformational PES of glycolic acid optimized at the B2PLYP-D3BJ/maug-cc-pVTZ(-dH) level. For transition states the imaginary frequency is also reported.

	**SSC (1)**		
C	-0.740950	0.774188	0.000000
H	-0.713257	1.420644	-0.880339
H	-0.713257	1.420644	0.880339
O	-1.897983	-0.020196	0.000000
H	-1.603970	-0.941524	0.000000
C	0.504024	-0.076447	0.000000
O	0.487861	-1.284885	0.000000
O	1.629062	0.659187	0.000000
H	2.376790	0.043172	0.000000
	**GAC (2)**		
C	-0.763878	-0.747888	0.181175
H	-0.807880	-1.090705	1.221142
H	-0.780811	-1.628504	-0.453956
O	-1.873382	0.049874	-0.160764
H	-1.760075	0.921367	0.231002
C	0.586824	-0.085358	0.010603
O	1.629506	-0.678170	-0.099316
O	0.499415	1.265293	0.054802
H	1.401336	1.609569	-0.023765
	**AAT (3)**		
C	0.703385	-0.824116	-0.036604
H	0.724304	-1.386288	-0.970301
H	0.694579	-1.529188	0.793964

O	1.796584	0.085348	0.060645
H	2.585967	-0.307339	-0.319221
C	-0.606060	-0.050198	-0.003970
O	-1.671166	-0.605802	0.022859
O	-0.480351	1.285507	-0.019539
H	0.469356	1.491170	-0.036484
	**ASC (4)**		
C	0.754520	0.736840	0.000000
H	0.720491	1.382692	0.882043
H	0.720491	1.382692	-0.882043
O	1.870502	-0.120441	0.000000
H	2.665459	0.418843	0.000000
C	-0.505916	-0.095676	0.000000
O	-0.577501	-1.293571	0.000000
O	-1.588959	0.722895	0.000000
H	-2.369477	0.150084	0.000000
	**AAC (5)**		
C	0.757223	-0.767948	0.052475
H	0.753204	-1.497130	-0.760646
H	0.776230	-1.323200	0.994328
O	1.827105	0.147312	-0.050004
H	2.650359	-0.337263	0.052605
C	-0.583582	-0.072367	0.004169
O	-1.623707	-0.685527	-0.032552
O	-0.507496	1.267429	0.022436
H	-1.421043	1.589911	-0.006597
	**SST (6)**		

C	-0.731417	-0.782202	0.000000
H	-0.703671	-1.427954	0.883835
H	-0.703671	-1.427954	-0.883835
O	-1.893244	-0.002017	0.000000
H	-1.598903	0.921041	0.000000
C	0.510404	0.089456	0.000000
O	0.452785	1.290694	0.000000
O	1.697072	-0.552006	0.000000
H	1.565183	-1.508236	0.000000

AST (7)

C	-0.746102	-0.742712	0.000000
H	-0.714032	-1.387742	0.885790
H	-0.714032	-1.387742	-0.885790
O	-1.870124	0.098843	0.000000
H	-2.661252	-0.446744	0.000000
C	0.513455	0.107876	0.000000
O	0.544632	1.301136	0.000000
O	1.664910	-0.621919	0.000000
H	1.472613	-1.567290	0.000000

TS12 $\omega_i = 90i$ cm^{-1}

C	0.84721800	-0.30003700	0.55477500
H	0.86968000	-1.37347200	0.73912700
H	1.06985900	0.22801100	1.47796900
O	1.83492000	0.09987600	-0.38019100
H	1.74113500	-0.43073800	-1.17703000
C	-0.54439100	0.12191200	0.10484500
O	-0.92131500	1.25827400	-0.01746200

O	-1.32730600	-0.95184900	-0.17059600
H	-2.18802700	-0.60546200	-0.45179300
**TS14** $\omega_i = 231i$ cm^{-1}			
C	0.75443300	-0.67292800	0.03317300
H	0.70571300	-1.41054300	-0.77110100
H	0.77243000	-1.22355000	0.97607600
O	1.86159300	0.18219700	-0.11956800
H	2.55634300	-0.09042800	0.48230600
C	-0.53334200	0.12374600	0.01032800
O	-0.64186700	1.31899700	0.02861500
O	-1.59122300	-0.72649200	-0.02496600
H	-2.38905700	-0.17799800	-0.02092600
**TS16** $\omega_i = 564i$ cm^{-1}			
C	0.70158900	-0.74842500	-0.03558600
H	0.65105600	-1.33485000	-0.95791300
H	0.63466100	-1.45636900	0.79511200
O	1.89590400	-0.01776700	0.04271600
H	1.65460800	0.91728400	-0.00383200
C	-0.51290300	0.14307600	-0.00714900
O	-0.44572200	1.34082900	-0.00103600
O	-1.70684400	-0.53519600	-0.08705200
H	-2.01914700	-0.79689500	0.78601800
**TS23** $\omega_i = 552i$ cm^{-1}			
C	-0.73259400	-0.75163800	-0.14770800
H	-0.76072600	-1.61655200	0.50810800
H	-0.80352800	-1.11540100	-1.17841800
O	-1.81628000	0.08142000	0.20007500

H	-1.79180000	0.85743600	-0.36875100
C	0.63001900	-0.09599400	-0.00801800
O	1.65113600	-0.70686700	0.11049400
O	0.60150600	1.28158100	-0.15175000
H	0.48060700	1.71123500	0.70286200

TS25 ω_i = 65i cm^{-1}

C	-0.85286200	-0.35230200	0.53286100
H	-1.00317900	0.13886000	1.49139600
H	-0.99437600	-1.42534600	0.65040500
O	-1.79452800	0.21992100	-0.36038700
H	-1.78756300	-0.28613800	-1.17852200
C	0.57847600	-0.12587100	0.07729300
O	1.34472400	-1.00274500	-0.24463000
O	0.90219400	1.18258300	0.06026300
H	1.81232100	1.24358800	-0.26617200

TS35 ω_i = 606i cm^{-1}

C	0.77188000	-0.63870100	-0.30800600
H	0.89793500	-0.64627500	-1.39546300
H	0.82402500	-1.66151000	0.06236300
O	1.71711200	0.20998400	0.32322000
H	2.59769700	-0.03045900	0.02462700
C	-0.61999200	-0.11079100	-0.02943400
O	-1.55335400	-0.81646000	0.22821000
O	-0.73679000	1.24606200	-0.21255000
H	-0.64673600	1.71850300	0.62207100

TS36 ω_i = 84i cm^{-1}

C	0.83750800	-0.16188300	0.61526100

H	0.92731300	-1.16282600	1.03286200
H	1.06957200	0.56614100	1.39259500
O	1.75776900	-0.09857200	-0.46432100
H	1.73052900	0.79417600	-0.82595800
C	-0.57100400	0.13086900	0.10801300
O	-0.97642700	1.25124800	-0.05306100
O	-1.34071700	-0.93485600	-0.21525600
H	-0.85143600	-1.75396700	-0.07803300

TS45 ω_i = 65i cm^{-1}

C	-0.85252600	-0.21519500	0.57741600
H	-1.07747400	0.44286000	1.41786400
H	-0.89703400	-1.25567500	0.89858900
O	-1.71659800	0.03011100	-0.52479600
H	-2.62439200	-0.06826100	-0.22554400
C	0.54961000	0.11892300	0.10371700
O	0.98155800	1.23448900	-0.03062700
O	1.26993000	-0.99487400	-0.15763300
H	2.13727400	-0.69910800	-0.47326000

TS47 ω_i = 534i cm^{-1}

C	-0.72237100	-0.67379300	-0.04170000
H	-0.65952800	-1.40250000	0.77399800
H	-0.69535300	-1.23810100	-0.97879200
O	-1.85357700	0.15481000	0.06495800
H	-2.63845800	-0.37709200	-0.09013600
C	0.53304900	0.16417400	-0.00981400
O	0.58396700	1.35436100	-0.00662300
O	1.66940000	-0.63270600	-0.08206200

H	1.93094900	-0.93631500	0.79382500

TS67 ω_i = 196i cm^{-1}

C	0.72218200	-0.67257200	0.07007600
H	0.65698400	-1.46049300	-0.68894400
H	0.74010000	-1.15862900	1.04988800
O	1.84695300	0.14008800	-0.14416500
H	2.56812200	-0.16226400	0.41161400
C	-0.54958900	0.16421200	0.01316000
O	-0.59519100	1.35716100	0.03518900
O	-1.69123600	-0.57846400	-0.03734700
H	-1.48496700	-1.51873000	-0.10139100
